# An enculturation-induced joy bias for emotion recognition in full-body-movement

**DOI:** 10.1038/s41598-025-24332-w

**Published:** 2025-10-23

**Authors:** Julia F. Christensen, Klaus Frieler, Meghedi Vartanian, Shahrzad Khorsandi, Fahima Farahi, Sina H. N. Yazdi, Susana Bravo Serra, Vincent Walsh

**Affiliations:** 1 Department of Cognitive Neuropsychology, Max Planck Institute for Empirical Aesthetics, Grüneburgweg 14, 60322 Frankfurt am Main, Germany; 2 Department of Neurology, Max Planck Institute for Human Cognitive and Brain Sciences, Leipzig, Germany; 3 Clinic for Cognitive Neurology, University of Leipzig Medical Center, Leipzig, Germany; 4 Shahrzad Dance Academy, Richmond, California, USA; 5 WiseWold.AI, Porto, Portugal; 6 Missis Bravo Films, Palma, Spain; 7 Institute for Cognitive Neuroscience, University College London, London, UK; 8 Max Planck Institute for Empirical Aesthetics, Frankfurt am Main, Germany

**Keywords:** Theory of constructed emotion, Basic emotions, Enculturation and acculturation, Cross-cultural, Full-body movement, Non-verbal communication, Expressivity, Iran, Europe, Persia, Dance, Iranian dance, Emotion recognition, Emotion labelling, Psychology, Human behaviour, Social behaviour

## Abstract

While emotional expression via the body is universal across cultures, *labelling* emotions into ‘emotion-word’ categories is not universal, but learned—especially in the West. Based on previous work using Western expressive gestures, we designed a video-stimuli library with emotionally expressive gestures from a non-WEIRD cultural tradition, which our participants had different levels of enculturation with. Stimuli consisted of 6-s-long sequences of Iranian social dance gestures, danced five-times each with different emotional expressivities, so that the *same* movement trajectories were used to express five ‘basic’ emotions (anger, fear, joy, sadness, neutrality). Across two experiments with 200 Iranian, English, and Southeast Asian participants (one pre-registered), we tested how enculturation modulated emotion perception from full-body movement. Using continuous measures of enculturation with Iranian and English culture, we found that categorical emotion *labelling* was modulated by English enculturation, while enculturation with Iranian culture produced a ‘joy bias’; a tendency to attribute joyful expressivity to the movements, in accordance with the joyful festive context in which these social dance gestures usually occur. These results evidence an effect of enculturation on emotion perception, in line with the theory of constructed emotion.

**Supplementary Information:**

The online version contains supplementary material available at 10.1038/s41598-025-24332-w.

## Introduction

Dance is a perversive human activity. All cultures, all over the world, engage in dancing. Dancing often happens within culturally relevant contexts like social gatherings, festive holiday events, during rites of passage, for meditation or other spiritual pursuits, or just for fun. On a basic perceptual level, when one person watches another person dancing, the observer perceives another social agent and their expressive full-body language. Within this work, we ask whether the comprehension of the expressivity of dance-based body language is modulated by enculturation, that is, prior experience with the culture that the dance originates from. We frame this central research question against the backdrop of current emotion theories, without pretending to conduct an exhaustive test of different emotion theories against each other.

Briefly, the traditional universal basic emotions account proposes that a small set of fixed, culturally universal ‘basic’ emotions exists, including joy, surprise, fear, disgust, anger, and sadness^1,2^. Yet, since the seminal studies by Ekman and colleagues, other work like that by Barret-Feldmann and colleagues has evidenced that there are no universal physiological nor neural activation patterns for specific “basic emotions” in the human brain and body^3–8^. These alternative, constructivist accounts of emotion, conversely, propose that experiencing emotions, be they one’s own, or those expressed by others, is a dynamic process, dependent on both socialization and enculturation processes^9^. To investigate the influence of enculturation on emotion recognition, we here loosely test constructivist approaches to emotion against the universal basic emotions account, by contrasting individuals’ experience of expressive body movement within a fairly culture-specific context: a dance tradition^10–13^.

### Dance in emotion research

Dance is an instance of expressive body-language *par excellence*^14–22^, and is, therefore, a valuable object of investigation in the domain of emotion research^14,23–30^. Traditionally, full-body emotion perception research has relied on videos showing individuals expressing every-day emotional ‘actions’, like punching a fist in anger, or jumping of joy^31–35^. Dance movements have also been used to investigate observer recognition of ‘basic emotions’ from full-body actions, e.g., using Indian classical dance, Bharatanatyam^36^ and Western classical ballet^15,16^. However, in recent years, a same-sequence/different-expressivity stimuli design has gained momentum in emotion research. Video clips of people walking, throwing, knocking, drinking, or waving in an, e.g., happy or sad way, are now routinely used as a more challenging emotion perception task^37–41^. This design avoids the often found ceiling effects with emotional-actions stimuli. Within this same-sequence/different-expressivity approach, it has been shown that subtle changes in kinematic parameters of movement execution convey different emotional expressivities to human observers (e.g., anger is characterized by brisk changes in acceleration, fearful movements are often jerky, while joyful movements are fluid, etc.)^18,42–46^.

Given the interconnections between dance practices and emotional experience, demonstrated by previous work^10,36,47,48^, we propose that dance lends itself particularly well to cross-cultural emotion perception research.

### Influence of culture on emotion perception from full-body movements

Recurring yearly festivities and events provide abundant opportunities for priors to be shaped with regards to the foods, songs, music, and dance of a culture—and with regards to their respective context-dependent expressivities^48^. Some dance styles of this world are mainly danced to express just one emotional valence, either positive or negative. Argentine and Uruguayan tango expresses mainly melancholy and nostalgic longing^49–53^. Many Middle Eastern fight dancers, like dancers of Palestinian *Dabkeh*, express positivity and a sense of pride, a will to conquer, prowess, and strength^54^. Brazilian *Samba de Roda* (‘circle Samba dance’) expresses joy, festive spirit, pride, and a will for freedom^55^. Conversely, other dance styles are used to express both positive and negative affect, depending on the dancers’ intention and the context. The 2000-year old syllabus of Indian classical dance^56^, *Bharatanatyam*, specifies how a large range of emotional expressions, positive and negative, and even complex story lines are to be expressed through dancing, and empirical work in experimental psychology confirms that observers can perceive these emotions in the movements^36,57^. Depending on context, the moves of the Maori *Haka* dance of New Zealand are used either to happily welcome guests, or to aggressively intimidate an enemy before a battle^58,59^. Western classical and romantic ballet practitioners convey emotions ranging from despair, loss, fear, sadness, nostalgia, and anger (e.g., in Jean Coralli’s ‘Giselle’ or Maurice Petipa’s ‘Swan Lake’), to happiness, wonder, love, and curiosity (e.g., in Maurice Petipa’s ‘The Nutcracker’ or ‘Cinderella’)^60–67^. All these dance styles are performed and practiced at social events such as yearly cultural festivities. They form a social communication system where the perception and the interpretation of their expressivity depends, in part, on an individuals’ enculturation. According to work in immigration psychology, *enculturation* is the early neurodevelopmental process that happens as children grow up and are exposed to the socio-cultural learning environment within a given cultural community^68,69^. Enculturation processes strongly influence peoples’ feelings of connectedness to traditions and arts of their culture^68,70–78^. This is also the case for the dance tradition featured in the present study: Iranian classical dance^10,12,13^.

### Iranian classical dance in empirical research

Iranian classical dance is a solo dance style mainly expressing joy and festive feelings^11,12,79^, for instance, at the yearly Yalda and Norouz festivities. It is mainly practiced in closed social settings, i.e., at birthdays, weddings, and other private gatherings. Its articulates various cultural mannerisms, including female coyness and male flirtatiousness^10,13^. Persian artforms are generally reflected in each other^11,80–83^, and this is also true for Iranian classical dance. This interconnectedness is shown figuratively in Persian Negargari paintings, and through fluid, intricate transitioning between movements in Iranian classical dance. Similarly, specific patterns and symmetries are very prevalent in Persian architecture and garden design (e.g., ornate frames that frame windows and doorways, that frame other parts of the building in return). Iranian dance retraces these patterns and symmetries in its positions; for instance, arms, and hands frame different parts of the face and the body^10,11,13^. Given its unique characteristics, Christensen et al.^10^ recently provided a detailed analysis of Iranian classical dance and proposed it as a valuable resource for empirical research. Importantly, for the present investigation, it is culturally very uncommon to express any negative emotional valence with Iranian classical dance gestures. Thus, according to the theory of constructed emotion, individuals enculturated with Iranian culture should struggle to recognize negative emotions in this dance genre.

### The present study and predictions

Our central research question was how the perception of emotional expressivities of dance depends on the observers’ level of enculturation with the culture of the dance style.

We predicted no difference between the two cultural groups (Iranian, English) in emotion recognition ability. This prediction builds on a previous study, in which the authors used an emotion recognition task with full-body videos of Indian Bharatanatyam, expressing seven basic emotions. No difference in emotion recognition between their groups of Indian and American participants was found (Hejmadi et al. 2001). We hypothesise that, instead, any differences would be a matter of degree. Due to the rise in the interconnectedness of today’s cultures, grown over the last decade via travel, immigration, and the internet, we expected any differences to be a matter of degree of *enculturation* (prediction 1). Therefore, in addition to participants’ self-identification as member of one cultural group or another (Iranian, English), we also administered screening measures of participants’ enculturation with Iranian and English culture. Enculturation and acculturation strongly influence peoples’ feelings of connectedness to traditions and arts^68,70–78^. While *enculturation* is the early neurodevelopmental process that happens as children grow up and are exposed to the socio-cultural learning environment within a given cultural community, *acculturation* is defined as the process occurring as an individual is immersed into new socio-cultural learning environments^68,69^. In experiment 1, 40 Iranian and 40 English participants filled in two recently developed screening tools in Farsi and English, that measure enculturation and acculturation^48^. These questionnaires were developed based on enculturation and acculturation measures used in immigration psychology^69,78,84–87^. For experiment 1, we included the *Cultural Traditions:* England vs Iran (CTQ-en/ir), the *Arts Engagement in Childhood:* England vs Iran (AECQ-en/ir) (Experiments 1). For experiment 2, we also administered the *Enculturation and Acculturation Quiz (EAQ-en/ir)* (experiment 2).

In line with the theory of constructed emotions, our second prediction was that individuals enculturated with Iranian cultural traditions, would *not* have any priors of Iranian classical dance movements expressive of sadness, fear, or anger, and therefore show a bias towards interpreting these movements as joyful, regardless of the dancer’s intended expressivity (and in accordance with the positively valenced context in which this body language usually occurs) (prediction 2). To strengthen this prediction we included a third cultural group in experiment 2 (in addition to 40 Iranian and 40 English participants), to test the impact of acculturation on emotion recognition. Thus, we selected 40 Southeast Asian participants, born and raised in England. This latter cultural group was, hence, not *enculturated* with English and Iranian culture, while being *acculturated* to English, but not to Iranian culture. Their responses were, therefore, expected to be more similar to the English than to the Iranians. Within the English cultural sphere, it is more common to attribute basic emotions like sadness, fear or anger to dance movements, while in Iran it is not. Therefore, we expected English and Southeast Asian participants to have a more accurate emotion recognition also for negative emotions.

Predictions were tested across two online experiments (one pre-registered). The first experiment, with 80 Iranian and English participants, allowed us to design an Iranian classical dance stimulus library (stimuli N = 100), and validate it with regards to emotion recognition accuracy (experiment 1; N = 80). The data from experiment 1 served also to confirm that the intended emotion by the dancer, was indeed perceived by the observers. The second experiment (pre-registered), with 120 participants, employed an optimized randomized block design with a reduced stimulus set (using the stimuli that had been recognized best in experiment 1) (stimuli N = 25), to replicate and expand the findings from the first experiment. Thus, in this study, we test how enculturation and acculturation processes modulate the perception of emotionally expressive full-body movements, as well as the verbal labelling of emotion categories. Our predictions are summarized in Table 1.

**Table 1:** Predictions.

Prediction 1: emotion recognition	
Emotion recognition is predicted to be low, and with no differences between the groups as in Hejmadi et al.^36^	
Prediction 2: enculturation and acculturation effects on emotion recognition	
Individuals enculturated with Iranian cultural traditions will not have any priors of Iranian classical dance movements expressive of sadness, fear, or anger, and therefore show a bias towards interpreting these movements as joyful, regardless of the dancer’s intended expressivity (and in accordance with the context in which this body language usually occurs—joyful)	

Across two experiments with 200 self-identified ‘Iranian’, ‘English’, and ‘Southeast Asian’ participants, we investigate how enculturation and acculturation modulate the perception of expressive body language, in a novel culturally-sensitive emotion recognition task with a newly created Iranian classical dance stimuli library.

## Results

### Results for prediction 1: emotion recognition

#### Results emotion recognition (experiment 1)

Participants’ emotion recognition with the full set of N = 100 stimuli was between 5 and 91.2% correct responses for individual stimuli (M = 41.6%; SD = 22.6%). A chi-square test revealed that all emotions had been recognized above chance level of 20% recognition rate (all ps < .001). See Table 2.

**Table 2:** Confusion matrix of emotion recognition accuracies in experiment 1.

Intended emotion by the dancer	% Decoded emotion by observers				
	Anger	Fear	Joy	Neutral	Sadness
Anger	**34.0**	16.1	19.8	22.7	7.4
Fear	11.0	**31.8**	16.9	21.3	19.0
Joy	6.4	6.3	**48.1**	23.9	15.3
Neutral	4.6	8.8	25.4	**38.0**	23.2
Sadness	3.9	6.6	12.1	21.4	**56.1**

N = 80. Experiment 1 participants’ emotion recognition accuracies and misclassifications, averaged over the scores of Iranian and English participants. Bold indicates correct replies (%). Emotion recognition for individual stimuli ranged between 5 and 91.2% correct responses (M = 41.6%; SD = 22.6).

The scores on participants’ enculturation measures CTQ-en, CTQ-ir, AECQ-en, and AECQ-ir differed significantly between the two cultural groups. English participants scored higher on the CTQ-en (m = 24.85; SE = 1.28), than the Iranians (m = 16.13; SE = 1.56; *p* < .001). Iranians scored higher on the CTQ-ir (m = 31.10; SE = 1.57), than the English (m = 0.00; SE = 0.00; *p* < .001) and they also scored higher on the AECQ-ir (m = 22.90; SE = 1.59), than the English (m = 1.60; SE = 0.78; *p* < .001). There was no difference between the groups of the AECQ-en between English (m = 9.98; SE = 1.32) and Iranian participants (m = 13.40; SE = 1.63; *p* = .107). The enculturation measures were only loosely intercorrelated (all rs < .45), except for the correlation between CTQ-ir and AECQ-ir (r = .85, *p* < .001). Only a few emotion recognition scores and questionnaire scores were loosely intercorrelated, posing no issue for the subsequent repeated measures ANOVA; fearful and sad (r = .333, *p* = .003), joy and neutral (r = − .233, *p* = .047), neutral and AECQ-ir (r = − .313, *p* = .005), sad and AECQ-en (r = .317, *p* = .004). All other rs < .217 and all ps > .065.

A repeated measures ANOVA was conducted, with the within-group factor emotion (anger, fear, joy, neutral, sad), the between group factor culture (Iranian, English), and the covariates CTQ-en, CTQ-ir, AECQ-en, and AECQ-ir. The dependent variable was participants’ percentage of correct responses.

Mauchly’s test of sphericity was significant, indicating that sphericity had been violated (*p* < .001). Thus, Greenhouse–Geisser correction was used in what follows. There was a main effect of emotion F(3.12, 230.69) = 3.419, *p* = .017, partial *η*^2^ = 0.044, no main effect of cultural background F(1, 74) = 0.058, *p* = .811, partial *η*^2^ = 0.001, and no interaction between emotion and cultural background F(3.12, 230.69) = 0.166, *p* = .358, partial *η*^2^ = 0.015. None of the interactions between emotion and the questionnaire scales were significant (all *p*s > .108), but there were main effects of AECQ-en F(1, 74) = 7.115, *p* = .009, partial *η*^*2*^ = 0.088 and of AECQ-ir F(1, 74) = 7.943, *p* = .006, partial *η*^2^ = 0.097. Šidák-adjusted pair-wise comparisons revealed that across all participants, joy had been recognized more accurately (m = 48.1%; SE = 1.8%; 95% CI [44.6%, 51.1%]) than anger (m = 34%; SE = 1.8%; 95% CI [30.6%, 37.4%]), fear (m = 31.1%; SE = 1.8%; 95% CI [28.4%, 35.3%]) and neutrality (m = 38%; SE = 1.8%; 95% CI [34.6%, 41.4%]) (all *p*s < .009), while sadness (m = 56.1%; SE = 1.8%; 95% CI [52.6%, 59.5%]) had been recognized over and above all other emotions (all *p*s < .001). There were no differences in emotion recognition accuracy between neutral and anger (*p* = .897) and neutral and fear (*p* = .195). Fear was recognized least well of all emotions – this pattern has previously been reported for full-body emotion stimuli^48,88^.

**Fig. 1:**
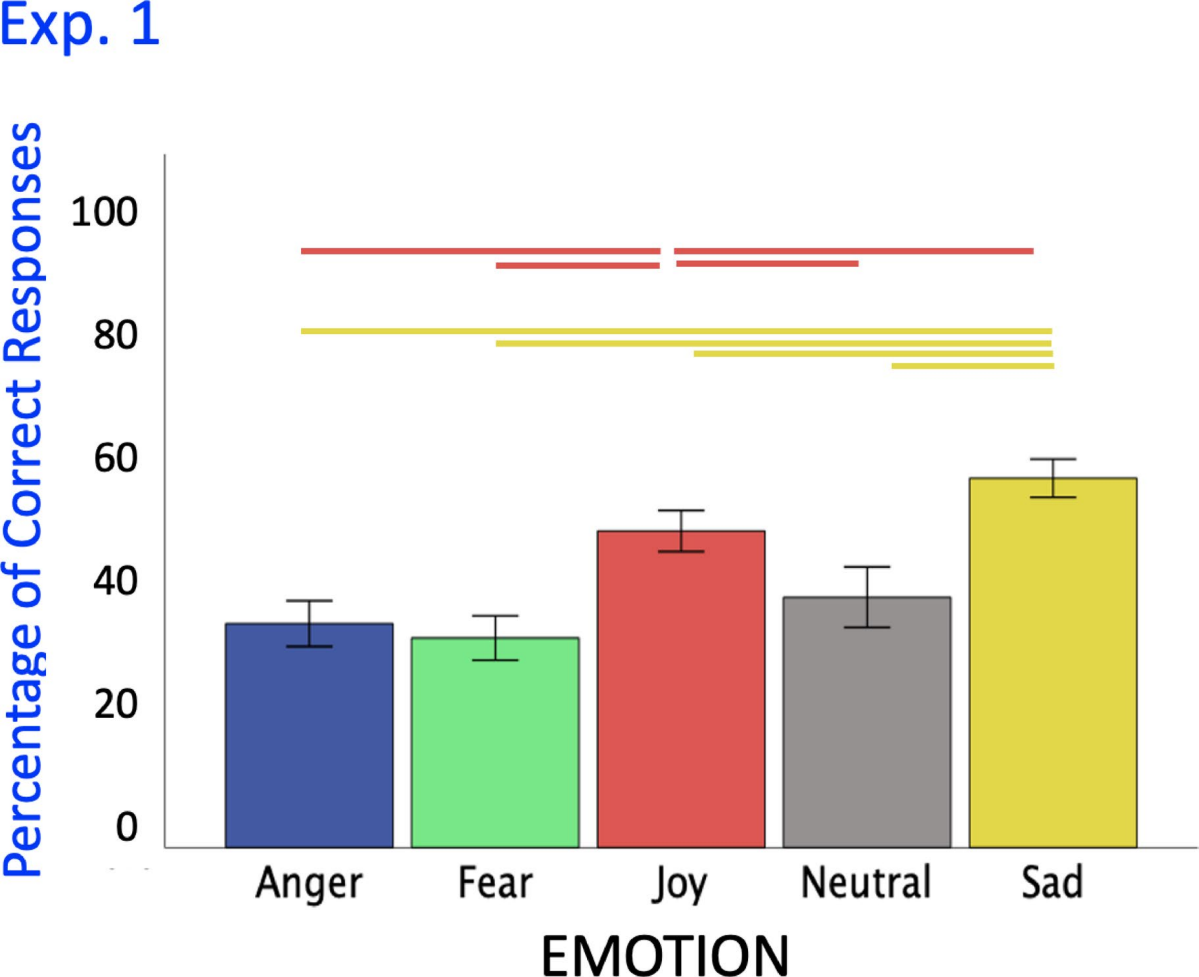
Results of experiment 1 participants’ percentage of accurate emotion recognition. *Note*: Experiment 1: N = 80 with 100 stimuli. Main effect of emotion on participants’ emotion recognition (%) (lines = all *p*s < .001). Joy (red) and sad (yellow) movements were recognized more accurately than angry (blue) and fearful (green) movements, but less accurately than neutral (grey), the latter been recognized over and above all others. A chi-square test revealed that all emotions had been recognized above chance (20%) (all *p*s < .001).

Although technically impossible, some participant IDs in the Iranian group appeared to have participated more than once. Upon inspection however, on average there were about 40–50% different values, and no two participants with the same prolific ID seemed to have identical data, so they likely were different participants. Nevertheless, we re-ran the analyses based on the first participation of each prolific ID (English participants = 40; Iranian participants N = 32) and the pattern of results was exactly the same, apart from differences in decimal places (supplementary material). See Fig. 1.

Following up the main effect of the AECQ-ir score, we conducted exploratory bivariate Pearson correlations which revealed that enculturation with Iranian arts in formative years of life had a trend effect to a positive correlation with emotion recognition for joyful movements (*p* = .075, r = .200) and negatively for neutral movements (*p* = .013, r = − .277). All other ps > .499.

#### Results emotion recognition (experiment 2)

In experiment 2, with the reduced set of the 25 best-recognized stimuli from experiment 1, participants’ emotion recognition rates ranged from 16 to 64% for individual stimuli (M = 38.3%, SD = 8.8%). A Chi-Square test revealed that participants had recognized all intended emotions above guessing probability of 20% (all *p*s < .001), except anger. See Table 3.

**Table 3:** Confusion matrix of emotion recognition accuracies in experiment 2.

Intended emotion by the dancer	% Decoded emotion by observers				
	Anger	Fear	Joy	Neutral	Sad
Anger	**22.8**	3.5	50.0	21.8	1.8
Fear	2.5	**24.8**	6.2	24.2	42.3
Joy	5.8	6.0	**61.0**	16.2	11.0
Neutral	2.5	15.7	25.0	**41.8**	15.0
Sad	1.7	20.8	12.2	24.5	**40.8**

N = 120. Experiment 2 emotion recognition accuracies and misclassifications, averaged over the scores of Iranian, Southeast Asian, and English participants. Bold indicates correct replies (%). Emotion recognition ranged from 16 to 64%, the average was = 38.3%, (SD = 8.8%).

Most of the scores on participants’ enculturation measures CTQ-en, CTQ-ir, AECQ-en, and AECQ-ir differed significantly between the three cultural groups. For descriptive purposes, we conducted a One-Way ANOVA with the three cultural groups as between-subjects factor and the three enculturation measures as dependent variables. English and Iranian participants differed between each other in their scores to both the England- and the Iran-related scales of all three questionnaires, each scoring higher on the scale of their respective culture. English and Southeast Asian participants differed between each other in all three scales for England-related cultural scales (English participants scoring higher), while they did not differ between each other for any of the Iran-related cultural scales. Southeast Asian participants did not differ from the Iranian participants in their scores to the England-related scales of the CTQ and the AECQ, but did show a significant difference for the EAQ-en; the Southeast Asian participants scoring higher than the Iranians. See Table 4 for detailed results.

**Table 4:** Enculturation questionnaire scores in experiment 2—culture group differences.

Measure	(A) Culture group	Mean (A)	*SE* (A)	(B) Culture group	Mean (B)	*SE* (B)	*p* value
CTQ-en	Southeast Asian	7.80	1.217	English	14.70	0.806	< .001**
				Iranian	9.23	0.995	.976
	English	14.70	0.806	Southeast Asian	7.80	1.217	< .001**
				Iranian	9.23	0.995	< .001**
	Iranian	9.23	0.995	Southeast Asian	7.80	1.217	.976
				English	14.70	0.806	< .001**
CTQ-ir	Southeast Asian	0.30	0.172	English	0.65	0.239	1.000
				Iranian	21.30	0.449	< .001**
	English	0.65	0.239	Southeast Asian	0.30	0.172	1.000
				Iranian	21.30	0.449	< .001**
	Iranian	21.30	0.449	Southeast Asian	0.30	0.172	< .001**
				English	0.65	0.239	< .001**
AECQ-en	Southeast Asian	3.23	0.693	English	6.73	0.779	.001**
				Iranian	3.65	0.564	1.000
	English	6.73	0.779	Southeast Asian	3.23	0.693	.001**
				Iranian	3.65	0.564	.006**
	Iranian	3.65	0.564	Southeast Asian	3.23	0.693	1.000
				English	6.73	0.779	.006**
AECQ-ir	Southeast Asian	1.38	0.337	English	1.48	0.349	1.000
				Iranian	9.10	0.727	< .001**
	English	1.48	0.349	Southeast Asian	1.38	0.337	1.000
				Iranian	9.10	0.727	< .001**
	Iranian	9.10	0.727	Southeast Asian	1.38	0.337	< .001**
				English	1.48	0.349	< .001**
EAQ-en	Southeast Asian	0.69	0.02	English	0.78	0.02	.026*
				Iranian	0.54	0.02	< .001**
	English	0.78	0.02	Southeast Asian	0.69	0.02	.026*
				Iranian	0.54	0.02	< .001**
	Iranian	0.54	0.02	Southeast Asian	0.69	0.02	< .001**
				English	0.78	0.02	< .001**
EAQ-ir	Southeast Asian	0.21	0.02	English	0.22	0.02	1.000
				Iranian	0.86	0.01	< .001**
	English	0.22	0.02	Southeast Asian	0.21	0.02	1.000
				Iranian	0.86	0.01	< .001**
	Iranian	0.86	0.01	Southeast Asian	0.21	0.02	< .001**
				English	0.22	0.02	< .001**

The questionnaires listed here are the *Cultural Traditions Questionnaire:* England versus Iran (CTQ-en/ir), the *Arts Engagement in Childhood Questionnaire:* England versus Iran (AECQ-en/ir) (Experiment 1) and the *Enculturation and Acculturation Quiz (EAQ-en/ir)* (Experiment 1 & 2). SE = Standard Error. *p* values are the results from a One-Way ANOVA. *p < .05; **p < .001.

A repeated measures ANOVA was conducted, with the within-group factor emotion (anger, fear, joy, neutral, sad), the between group factor culture (Iranian, English, Southeast Asian), and the covariates CTQ-en, CTQ-ir, AECQ-en, AECQ-ir and the new scales EAQ-en and EAQ-ir. The dependent variable was percentage of correct responses.

**Fig. 2:**
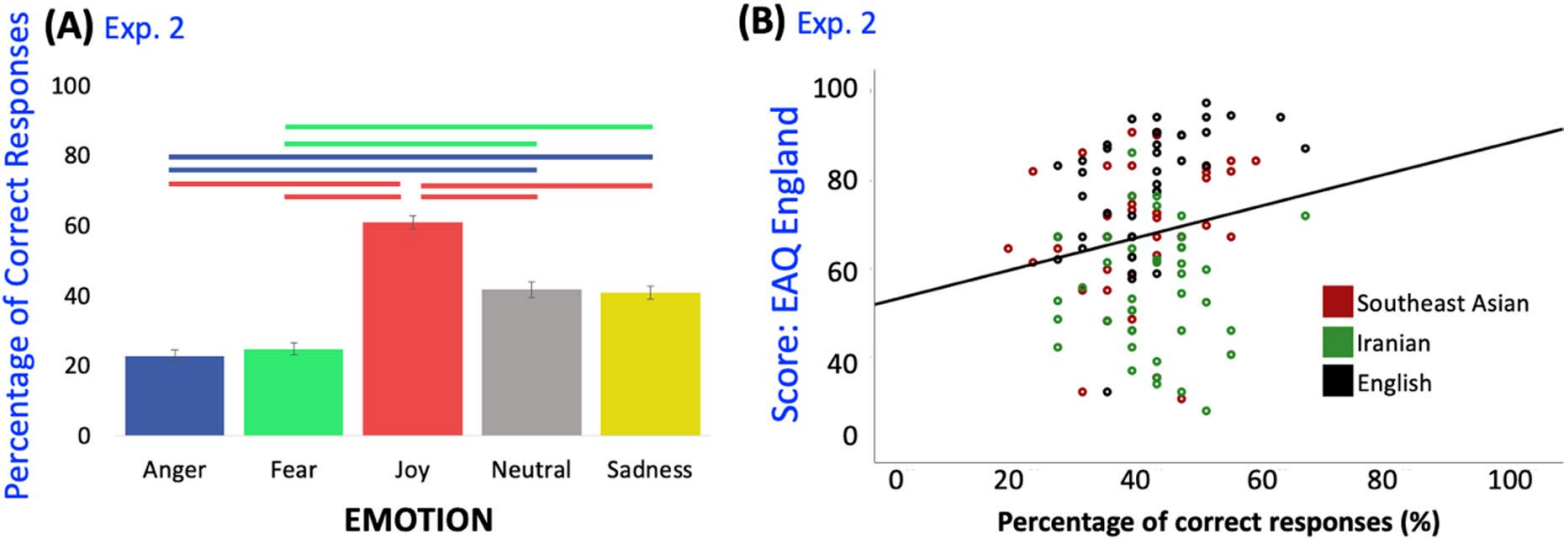
Results of emotion recognition task in experiment 2. *Note*: Experiment 2. N = 120; 40 Iranians, 40 English, 40 South-East Asian participants; with 25 stimuli. (**A**) The graph illustrates the main effect (*p* < .001) of intended emotion on participants’ emotion recognition rates (%) and Šidák-adjusted pair-wise comparisons between the emotions. Horizontal lines indicate significant differences, all *p*s < .001. A Chi-Square test revealed that all intended emotions except anger were recognized above guessing probability of 20% (all *p*s < .001). (**B**) Pearson correlations showed that knowledge of English culture (where the expression of negative emotions in dance is common) correlated positively with overall emotion recognition (*p* = .046, r = .182), and with emotion recognition for fearful movements (*p* = .01, r = .236) and for sad movements (*p* = .031, r = .197).

Mauchly’s test of sphericity was significant, indicating that sphericity had been violated (*p* < .001). Thus, Greenhouse–Geisser correction was used in what follows. There was a main effect of emotion (F(3.59, 398.17) = 6.127, *p* < .001, partial *η*^2^ = 0.052), no main effect of culture (F(2, 111) = 0.574, *p* = .565, partial *η*^2^ = 0.010), and no interaction between emotion and culture (F(7.17, 398.17) = 1.316, *p* = .233, partial *η*^2^ = 0.023). However, there was a significant interaction between emotion and one of the enculturation scales, the CTQ-ir (F(3.59, 398.17) = 2.713, *p* = .035, partial *η*^2^ = 0.024). None of the other interactions between emotion and the questionnaire scales were significant (all *p*s > .10), nor their main effects (all *p*s > .163), except for the new scale EAQ-en which was F(1, 111) = 9.141, *p* = .003, *η*^2^ = 0.076. Šidák-adjusted pair-wise comparisons revealed that significant differences in emotion recognition between all emotions (all *p*s < .001), except between sadness (m = 40.8%; SE = 1.9%; 95% CI [37.1%, 44.6%]) and neutrality (m = 41.8%; SE = 2.2%; 95% CI [37.4%, 46.2%]) that were recognized equally well (at about 40%; *p* = 1), nor between fear (m = 24.8%; SE = 1.7%; 95% CI [21.5%, 28.2%]) and anger (m = 22.8%; SE = 1.9%; 95% CI [19.1%, 26.6%]) (at about 23%; *p* = .993). Joy was the most easily recognized emotion (m = 61.0%; SE = 1.9%; 95% CI [57.2%, 64.8%]) followed by neutral and sadness, with anger and fear at the bottom, just above guessing probability level. See Fig. 2A.

Following up the interaction between emotion and the CTQ-ir score and the main effect of the EAQ-en, bivariate Pearson correlations were conducted with the total emotion recognition score and the individual emotions. Enculturation with Iranian cultural traditions (CTQ-ir) did not correlate significantly with any of the emotion recognition scores (all *p*s > .084), although emotion recognition for joyful movements was trending towards significance (*p* = .084, r = .158). Knowledge of English culture correlated positively with overall emotion recognition (*p* = .046, r = .182), and with emotion recognition for fearful movements (*p* = .01, r = .236) and for sad movements (*p* = .031, r = .197). See Fig. 2B. Šidák-corrected pair-wise comparisons showed a few differences within and between the cultural groups (using the grouping variable ‘culture’). For Southeast Asian participants, joy (m = 60.1%; SE = 1%; 95% CI [43.6%, 76.7%]) was recognized above fear (m = 25.1%; SE = 1%; 95% CI [10.7%, 39.5%]) (*p* = .044) and neutrality (m = 19.8%; SE = 1%; 95% CI [8%, 38.9%]) (*p* = .010). For English participants, the same differences were observed, between joy (m = 58.5%; SE = 1%; 95% CI [42%, 74.9%]) and fear (m = 22.3%; SE = 1%; 95% CI [8%, 36.6%]) (*p* = .032), and joy and neutrality (m = 18.5%; SE = 1%; 95% CI [− 0.4%, 37.5%]) (*p* = .010). For Iranians, however, there was only one difference, between emotion recognition for angry movements (m = 5.8%; SE = 15.2%; 95% CI [− 24.4%, 35.9%]) had been recognized better than neutral ones (m = 87.1%; SE = 17.8%; 95% CI [51.9%, 122.4%]) (*p* = .011). Between the groups, only for neutral movements a difference was observed. Iranians recognized neutral movements (m = 87.1%; SE = 17.8%; 95% CI [51.9%, 122.4%]) better than Southeast Asian participants (m = 19.8%; SE = 1%; 95% CI [8%, 38.9%]) (*p* = .039) and than English participants (m = 18.5%; SE = 1%; 95% CI [− 0.4%, 37.5%]) (*p* = .033).

### Results for prediction 2: enculturation effects on emotion recognition

#### Experiment 1

We next explored a possible “joy-bias” in participants’ interpretation of the stimuli as a function of the cultural groups (as defined by the Prolific® filter). A chi-squared test was conducted, which revealed that there was an overall bias for attributing joy (24.5%), neutral (25.5%), or sad (24.2%) emotion to the movements, (χ^2^(4) = 673.4, *p* < .001). However, this bias was slightly different across the cultural backgrounds (χ^2^(4) = 45.2, *p* < .001, Cramer’s V = − 0.08). When comparing English and Iranian participants Iranian participants were found to attribute joy more frequently than all other emotions (27.6%), while the English attributed neutral (26.3%), and sadness (25.2%) more often, and joy less frequently (21.3%). The alluvial plot in Fig. 2 sets out these differences.

Next, we defined the “joy bias”, for each participant separately, as the percentage of attributed joy. Pearson’s correlations between the joy bias and the two enculturation scales administered in Experiment 1 (CTQ-en and CTQ-ir; AECQ-en, and AECQ-ir) revealed that enculturation modulated the joy bias. The enculturation metrics with Iranian culture showed significant positive correlations, for arts engagement with Iranian arts in childhood (AECQ-ir; r = .39, *p* < .001), and practice of Iranian cultural traditions (CTQ-ir; r = .39, *p* < .001), whereas no significant correlations for the English enculturation metrics could be found. See Table 5.

**Table 5:** Correlations between “joy bias” and enculturation-acculturation scores.

Predictor	r	t	*p*
Arts engagement English (AECQ England)	.21	1.78	.08^t^
Arts engagement Iranian (AECQ Iran)	.39	3.57	< .001**
Tradition English (CTQ England)	− .13	− 1.07	.29
Tradition Iranian (CTQ Iran)	.39	3.49	< .001**

N = 120. Experiment 2. Correlations between “joy-bias” and enculturation scores. The “joy bias” is a metric, for each participant separately, showing the percentage of attributed joy. Cultural Traditions Questionnaire: England versus Iran (CTQ-en/ir) and the Arts Engagement in Childhood Questionnaire: England versus Iran (AECQ-en/ir). *p < .05, **p < .001, t = marginal effect.

#### Experiment 2

We next explored this “joy-bias” in participants of Experiment 2. A chi-squared test was conducted, which revealed that there was an overall bias for attributing joy to the movements, (χ^2^(4) = 535.07, *p* < .001). Again, this bias was slightly different across the cultural backgrounds (χ^2^(8) = 13.746, *p* = .09, Cramer’s V = 0.05). When comparing only English and Iranian participants (leaving out the Southeast Asian group from the analysis), Iranian participants were found to attribute joy more frequently than all other emotions, while the English attributed anger, fear, and sadness more often, and joy less frequently (χ^2^(4) = 11.903, *p* = .02, Cramer’s V = 0.07). Comparing joy vs. all other emotions, Iranians chose joy 37% of the time, the English 28%, and the Southeast Asian participants about 30%.

Next, we defined the “joy bias”, for each participant separately, as the percentage of attributed joy. Pearson’s correlations between the joy bias and the three enculturation scales (EAQ-en and EAQ-ir; CTQ-en and CTQ-ir; AECQ-en and AECQ-ir) again revealed that enculturation modulated the joy bias. Knowledge-based enculturation with English traditions (EAQ-en score) correlated *negatively* with a bias to attribute joy to the movements (r = − .240, *p* = .01), while the enculturation metrics with Iranian culture showed a positive correlation, for arts engagement with Iranian arts in childhood (AECQ-ir; r = .210, *p* = .02), practice of Iranian cultural traditions (CTQ-ir; r = .290, *p* < .001), and knowledge-based enculturation with Iranian traditions (EAQ-ir; r = .170, *p* = .07, marginal effect). See Table 6.

**Table 6:** Correlations between “joy bias” and enculturation-acculturation scores.

Predictor	r	t	*p*
AEQ English traditions	− .24	− 2.63	.01*
AEQ score Iranian traditions	.17	1.84	.07^t^
Arts engagement English (AECQ England)	.09	0.95	.34
Arts engagement Iranian (AECQ Iran)	.21	2.28	.02*
Tradition English (CTQ England)	.10	1.06	.29
Tradition Iranian (CTQ Iran)	.29	3.24	.00**

N = 120. Experiment 2. Correlations between “joy-bias” and enculturation scores. The “joy bias” is a metric, for each participant separately, showing the percentage of attributed joy. Cultural Traditions Questionnaire: England versus Iran (CTQ-en/ir), the Arts Engagement in Childhood Questionnaire: England versus Iran (AECQ-en/ir) and the Enculturation and Acculturation Quiz: England versus Iran (EAQ-en/ir). *p < .03, **p < .001, t = marginal effect.

To investigate the enculturation and acculturation effects further, correlations of the overall emotion recognition accuracy per participant with the six cultural variables was inspected. Only the English knowledge-based enculturation measure (EAQ-en) correlated positively with overall emotion recognition accuracy (r = .18, *p* = .05), as predicted by the assumption that attributing basic emotions to dance is culturally more English than Iranian. See Table 7.

**Table 7:** Emotion recognition accuracy correlated with cultural background variables.

Predictor	r	CI	t	*p*
Tradition English (CTQ England)	− .04	[− 0.22, 0.14]	− 0.46	.65
Tradition Iranian (CTQ Iran)	.05	[− 0.13, 0.22]	0.49	.62
Arts engagement English (AECQ England)	.00	[− 0.18, 0.18]	0.00	1.00
Arts engagement Iranian (AECQ Iran)	.01	[− 0.17, 0.19]	0.09	.93
AEQ English Traditions	.18	[0.00, 0.35]	2.01	.05*
AEQ Iranian Traditions	.13	[− 0.05, 0.31]	1.47	.14
Combined English	.06	[− 0.12, 0.24]	0.67	.50
Combined Iranian	.07	[− 0.11, 0.24]	0.73	.46

Correlations and confidence intervals of emotion recognition with six enculturation measures: Cultural Traditions Questionnaire: England versus Iran (CTQ-en/ir), the Arts Engagement in Childhood Questionnaire: England versus Iran (AECQ-en/ir) and the Enculturation and Acculturation Quiz: England versus Iran (EAQ-en/ir). *p < .05. After summing, the questionnaires were aggregated into ‘combined scores’.

## Discussion

Across two experiments, we tested the influence of enculturation on emotion recognition from full-body movements, and in so doing, we also loosely tested the basic emotion account against constructivist approaches to emotion. We found an influence of enculturation (not of culture), and some evidence that endorses constructivist approaches to emotion.

First, with regards to emotion recognition of the 5 basic emotions, in experiment 1, sadness had been recognized best of all emotions, followed by joyful movements, with no differences in emotion recognition for anger, fear and neutral movements, but all emotions had been recognized above chance-level of 20%. Fear was recognized least well of all emotions—this pattern has previously been reported for fear in full-body emotion stimuli^48,88^.

Second, emotion recognition of basic emotions expressed in dance movements was modulated by knowledge of English culture (EAQ-en). This is in line with prediction 1 and 2 in that it shows an enculturation effect. As set out in the introduction, Western dance styles like ballet are often used to express more than one emotional intention, including also negative emotional states like anger, fear and sadness—while such expressivities are not common in Iranian dance styles. Hence, enculturation with English culture, where these expressivities in dance are common, may explain this result.

Third, in experiment 2, the pattern was similar to experiment 1, just that joy had been recognized above all others, followed by sad movements. Neutral movements were recognized above fearful and angry movements, but there was no difference in emotion recognition between neutral and sad movements, nor between angry and fearful movements. This pattern of results is very similar to those found in a study with Western contemporary dance movements of 5 emotional expressivities^88^.

**Fig. 3:**
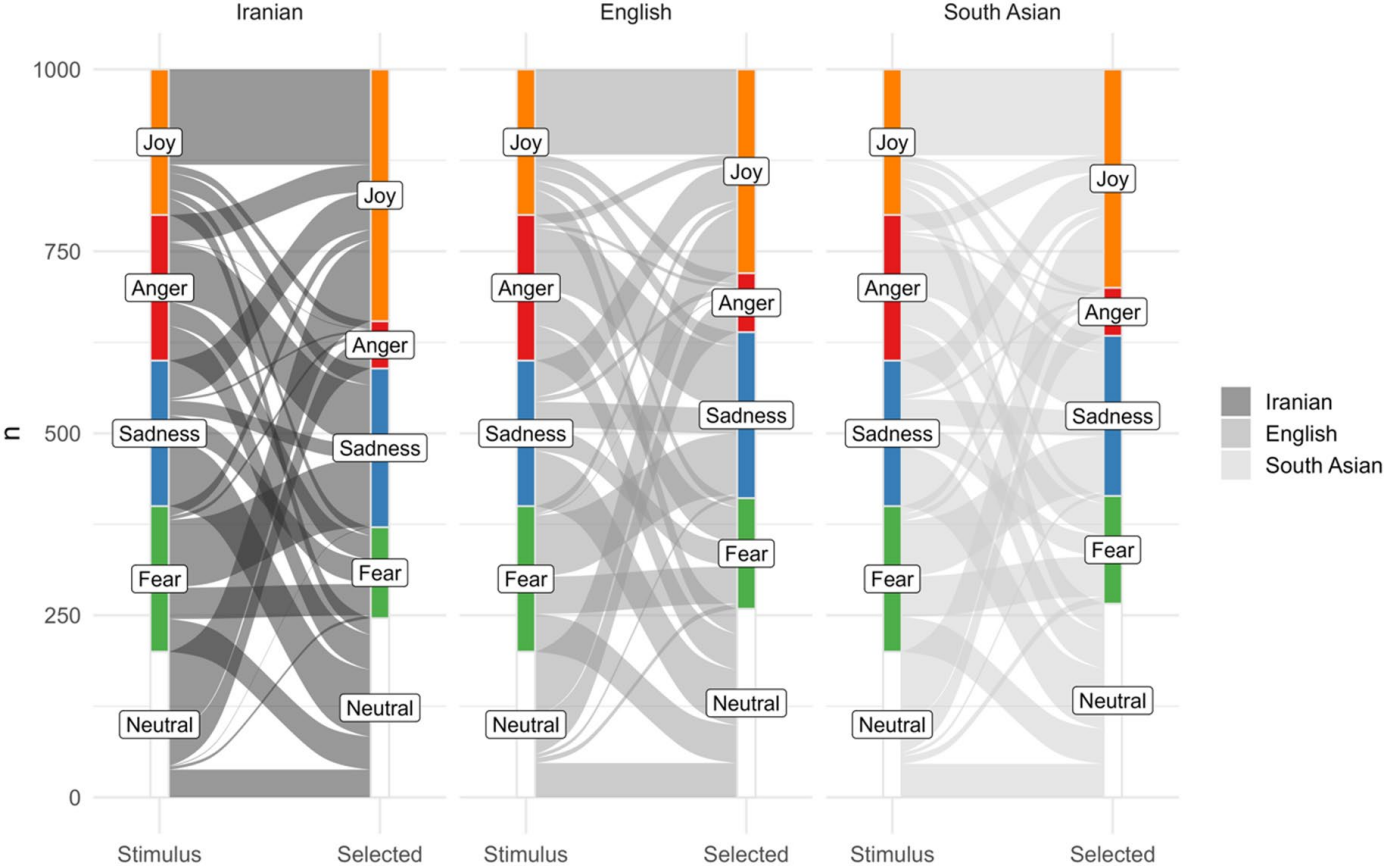
Alluvial plot emotion recognition and a function of cultural group. *Note*: The quantities of the classifications and misclassifications can be observed in the confusion matrices in Tables 2 and 3. Within the alluvial plot, these (mis)classifications are represented as the wavy and straight lines between emotions. Joy is represented as the colour orange. Iranians have selected joy (see x-axis) more frequently than it was intended for the stimulus (i.e., the orange line on the right is longer than the orange line on the left. All three groups selected anger very rarely—which is shown as a very short red bar on the right, as compared to the intended emotion for the stimulus (bar on the left).

Fourth, inspection of the confusion matrices (Tables 2 and 3), and the alluvial plot (Fig. 3), shows that there were some misclassifications of the emotions, even if the general emotion recognition rate was above chance level. Regardless of culture group, the many misclassifications of movements as ‘neutral’ could reflect participants’ inability to artificially classify full-body movements to any of the emotions prescribed by the forced-choice task (anger, fear, joy, sadness). Besides, usually anger, joy and fear are considered ‘high arousal emotions’, while sadness and neutrality are considered ‘low’ in arousal^89,90^. High arousal emotions are characterized by quick movements, which may explain why anger and joy are often confused in emotion recognition tasks; an effect that we also found in this dataset of Iranian dance. However, interestingly, the high arousal emotion fear was often confused with the low arousal emotion sadness, and joy (high arousal) with neutrality (no arousal). These results suggest that the same-sequence approach to stimuli materials (see the methods section for more details), may add new possibilities to emotion research, as this allows to study the contribution of intended emotional expressivity, regardless of the use of specific emotional actions to emotion recognition.

Fifth, across both experiments we detected another result that was in line with prediction 2. Iranians classified more stimuli as ‘joyful’ than English participants did, and the more individuals were enculturated with Iranian arts, traditions, and knowledge, the stronger was this joy bias. Importantly, this effect was a matter of *degree* of enculturation with Iranian culture and not found, when applying the self-ascribed *culture group* as a grouping variable in the analyses. These results highlight again the assumptions of constructivist theories^91^ that see emotional experience as the result of an experience-based learning process that is not categorical (‘culture group’), but fluid (‘enculturation’); in our study, enculturation modulated visual perception of expressive gestures^68,70–78^. Children, all over the world, spontaneously engage in dancing, and when they do, their expression is usually of pure joy. It may be a specific, perhaps particularly WEIRD^92^ enculturation process (which teaches children early on, that dance can express anger, fear or sadness), that alienates individuals from that ‘natural’ inclination to express (and understand) expressions of joy from dance. Hence, *not* having a joy-bias when doing a dance-based emotion recognition task may, in fact, be quite WEIRD.

Of course, there are limits to the generalisability of the findings reported in this study. We used only one dancer model, only one dance style. We believe that, this is a solid first assessment of expressive emotional Iranian dance movements, given the complexities associated with studying this dance style and finding dancers who can portray it authentically. We relied on a dance expert who was specifically trained in emotional expressivity, and experiment 1 served as a validation experiment. We saw that the intended emotions by the dancer were recognized above chance by the observers. Experiment 2, with a different set of participants replicated these findings. Hence, we take this as solid evidence that the intended emotions were indeed recognizable above chance level. This particular dance genre is under risk of underdevelopment and loss due to restrictions on dance in its country of origin^93–96^. Hence, this dance style is not perceived in an unbiased way by Iranians and many diverging views on the dance style are likely^12^. A strength of this study is that it included non-WEIRD participants in this study^92^, Iranians (and Southeast Asians; the latter born and raised in the UK). However, given the restrictions on dance in Iran and given the fact that Prolific is not accessible from within Iran due to international sanctions, our Iranian sample consisted of individuals living in diaspora. This could have led to a different set of answers than would potentially have been obtained if the data had been collected inside Iran. However, to our knowledge, there is currently no safe way to collect such data on dance within Iran in a sample that includes both men and women. We undertook efforts to describe the enculturation and acculturation with Iranian culture in a very granular way by means of our culture questionnaires. The fact that we did find a joy-bias in line with our predictions suggests that our Iranian sample retained sufficient cultural familiarity for this bias to emerge.

Dance practices are a mirror of the culture that surrounds it^47^, and our study shows how the perception of expressivity in full-body movements is modulated by enculturation; in this case, illustrated by a joy-bias, a tendency to attribute the emotion of ‘joy’ to dance movements in accordance to the cultural context that the dance originates from. The past centuries have seen many misunderstandings with regards to the interpretation of the dances of this world, with the most problematic and most documented instances being the orientalist interpretations of Middle-, Central- and Far-Eastern dance styles by Western travellers. Iran is one of the countries, where dancing in public remains heavily restricted, especially, for women, since the Islamic Revolution in 1979 due to ‘decency’ concerns^10,95,96^.

Much research in the past two decades has evidenced that dancing is ‘not just for sex’^17^ and has important positive psychobiological and cognitive effects for individuals^97,98^, along with social, cultural and regulative effects for societies^99–104^, thus, contributing to health of individuals and societies^105^. For good reasons, the UN Declaration of Human Rights^106^ has explicitly enshrined the freedom of expression through the arts in article 27 of the declaration, and this includes dance.

## Method

Experiment 1 was registered with the University College London Data Protection Officer; risk assessment and ethical approval was obtained (ICN-PWB-20-6-19A). Experiment 2 was covered under the umbrella approval of the Ethics Council of the Max Planck Society (Nr. 2017_12). The research complied with the UK Data Protection Act 1988, General Data Protection Regulation (GDPR). For the experiments (performed online via the platform Prolific®^107,108^), informed consent was obtained from all participants online through a tick-box system. All methods were performed in accordance with the relevant guidelines and regulations, and in accordance with the Declaration of Helsinki^109^. Experiment 2 was pre-registered on the OSF as part of a larger project: https://osf.io/ewp7c/overview.

Data from all experiments is available on the OSF: https://osf.io/2evgt/overview.

### Participants (experiments 1 and 2)

#### Participants (dancer model)

One dancer and choreographer with 30 years of international professional dance experience, who trained in Iranian–Persian dance as well as Modern dance. During her college education in dance, she also had some classical ballet technique training. Besides, she is the author of books and scholarly articles surrounding Iranian classical dance^10,13,93,94^.

#### Participants (experiments 1)

Eighty participants took part in the online survey; 40 Iranian participants (27 male; age all: m = 29.10, SD = 8.70), 40 English participants (18 male; age all: m = 34.12, SD = 7.40). See Table 8.

**Table 8:** Sample characteristics experiment 1.

	Culture	
	Iranian participants frequency (%)	English participants frequency (%)
Gender		
Female	32.5	55
Male	67.5	45
First language		
English	2.5	100
Farsi	87.5	–
German	2.5	–
French	2.5	–
Other	5	–
Location of life (first 12 years)		
UK	7.5	97.5
Iran	77.5	–
Europe	5	2.5
US America	2.5	–
Other	7.5	–
Professional dancer?		
Yes	2.5	–
No	97.5	100
Hobby dancer?		
Yes	57.5	10
No	42.5	90
Dance styles (hobby)		
Social dances	37.9	13
Persian dance	62.1	0.6
Contemporary	9.9	6.8
Ballet	4.3	6.8
Folkloric	23	–
Tap dance	5.6	1.9
African	1.9	1.2
Latin	16.8	3.1
Other	8.7	5.6
I don’t dance	27.3	75.8

N = 80.

The online questionnaire included a question ‘did you have technical difficulties with the survey (e.g., the videos didn’t play)?’ and responses were given on a 5-point scale (1 = not at all; 5 = a lot). Any participants who rated 3 or over were eliminated. This happened several times, however, we do not have any count of how many they were, as we simply tested until we had 40 participants of each group with ratings of 1 or 2 on this question.

#### Participants (experiment 2)

A total of 120 participants took part in Experiment 2; 40 Iranian participants (20 male; age all: m = 31.2; SD = 7.80), 40 English participants (20 male; age all: m = 32.6; SD = 8.94), and 40 Southeast Asian participants (20 male; age all: m = 31.5; SD = 8.99). This data was collected in May–June 2023. See Table 9.

**Table 9:** Sample characteristics experiment 2.

Baseline characteristic	Iranian	English (in England)	Southeast Asian (in England)
Age	31.18 (7.80) range = 39	32.25 (8.94) range = 31	31.45 (8.99) range = 39
Gender			
Female	19 (47.50%)	19 (47.50%)	20 (50.00%)
Male	20 (50.00%)	20 (50.00%)	20 (50.00%)
Prefer not to say	1 (2.50%)	1 (2.50%)	0 (0%)
Education			
Did not complete school	0 (0%)	0 (0%)	0 (0%)
Completed school up to age of 16 (e.g., GCSE)	0 (0%)	3 (7.50%)	1 (2.50%)
Completed further education (e.g., A-level, diploma)	2 (5.00%)	5 (12.50%)	9 (22.50%)
Some university or professional qualification	3 (7.50%)	5 (12.50%)	1 (2.50%)
Completed undergraduate or professional qualification	12 (30.00%)	16 (40.00%)	21 (52.50%)
Completed postgraduate degree (e.g., MA, PhD)	23 (57.50%)	11 (27.50%)	8 (20.00%)
Prefer not to say	0 (0%)	0 (0%)	0 (0%)
Total years of education	18.00 (5.51) range = 25	16.75 (2.51) range = 10	16.95 (3.23) range = 25
Lived first 12 years of life			
Iran	35 (87.50%)	0 (0%)	0 (0%)
United Kingdom	2 (5.00%)	37 (92.50%)	40 (100%)
Denmark	0 (0%)	1 (2.50%)	0 (0%)
Estonia	0 (0%)	1 (2.50%)	0 (0%)
Germany	0 (0%)	1 (2.50%)	0 (0%)
Emirates	1 (2.50%)	0 (0%)	0 (0%)
Netherlands	1 (2.50%)	0 (0%)	0 (0%)
South Africa	1 (2.50%)	0 (0%)	0 (0%)
Mother tongue			
Farsi	38 (95.00%)	0 (0%)	0 (0%)
English	1 (2.50%)	37 (92.50%)	28 (70.00%)
Bengali	0 (0%)	0 (0%)	3 (7.50%)
Gujarati	0 (0%)	0 (0%)	4 (10.00%)
Punjabi	0 (0%)	0 (0%)	3 (7.50%)
Other	1 (2.50%)	3 (7.50%)	2 (5.00%)
Multi-lingualism			
1 additional spoken language	27 (67.50%)	31 (77.50%)	35 (87.50%)
2 additional spoken languages	9 (22.50%)	6 (15.00%)	4 (10.00%)
3 or more additional spoken languages	4 (10.00%)	3 (7.50%)	1 (2.50%)
Languages spoken			
Arabic	(0%)	2 (1.67%)	1 (0.83%)
Bengali	(0%)	(0%)	5 (4.17%)
Cantonese	(0%)	(0%)	4 (3.33%)
English	4 (3.33%)	37 (30.83%)	12 (10.00%)
German	4 (3.33%)	2 (1.67%)	1 (0.83%)
Italian	4 (3.33%)	4 (3.33%)	(0%)
Punjabi	(0%)	(0%)	5 (4.17%)
Spanish	9 (7.50%)	(0%)	(0%)
Welsh	5 (4.17%)	(0%)	(0%)
Other	28 (23.33%)	13 (10.83%)	19 (15.83%)
Formal dance experience			
Yes	6 (15.00%)	11 (27.50%)	1 (2.50%)
No	34 (85.00%)	29 (72.50%)	39 (97.50%)
Average years of dance experience	1.58 (6.30)	2.13 (4.22) range = 17	0.13 (0.79) range = 5
Dance styles			
Ballet	1 (2.50%)	9 (22.50%)	2 (5.00%)
Disco (free dancing in the club)	1 (2.50%)	3 (7.50%)	1 (2.50%)
Hip hop and other street dance styles	0 (0%)	2 (5.00%)	1 (2.50%)
Jazz dance	0 (0%)	3 (7.50%)	0 (0%)
Modern dance/contemporary	1 (2.50%)	5 (12.50%)	0 (0%)
Persian dance	13 (32.50%)	0 (0%)	0 (0%)
Tap	0 (0%)	5 (12.50%)	0 (0%)
Other	7 (17.50%)	2 (5.00%)	1 (2.50%)
Social dance enjoyment			
Never	7 (17.50%)	5 (12.50%)	23 (57.50%)
Two or three times per week	4 (10.00%)	2 (5.00%)	0 (0%)
Once per week	3 (7.50%)	7 (17.50%)	2 (5.00%)
Once per fortnight	5 (12.50%)	2 (5.00%)	1 (2.50%)
Once per month	10 (25.00%)	6 (15.00%)	2 (5.00%)
Every couple of months	2 (5.00%)	8 (20.00%)	5 (12.50%)
A few times a year	9 (22.50%)	10 (25.00%)	7 (17.50%)
Iranian dance			
How important is Iranian dance for you?	73.68 (20.85)	26.18 (20.94)	26.58 (26.45)
Task interest			
How much did you enjoy this task?	3.43 (0.68)	3.67 (0.64)	3.42 (0.76)

N = 120. Mother tongue: Category “Other” summarizes languages that were referenced as a native language by less than two participants. The category includes Chinese, Danish, Dutch, Estonian, French, and Urdu. “Languages Spoken” includes languages spoken additionally to the native language. The category “Other” summarizes languages that are spoken by less than three participants and includes: Afrikaans, Catalan, Chinese, Czech, Danish, Dutch, Estonian, Farsi, French, Greek, Gujarati, Hebrew, Korean, Kurdish, Mandarin, Polish, Romanian, Russian, Swedish, Tamil, Thai, Turkish, and Urdu. Percentages are displayed as a fraction of the total sample size of n = 120. Dance styles: Category “Other” summarizes dance styles that were mentioned by two or less participants. Includes: African Dances, Argentine Tango, Belly Dance, Burlesque/Lyrical Dance, Folklore, Salsa/Bachata and Indian Dances.

As in experiment 1, the online questionnaire included a question ‘did you have technical difficulties with the survey (e.g., the videos didn’t play)?’ and responses were given on a 5-point scale (1 = not at all; 5 = a lot). We tested until we had 40 participants of each group with ratings of 1 or 2 on this question.

### Stimuli

#### Stimuli (experiment 1)

**Fig. 4:**
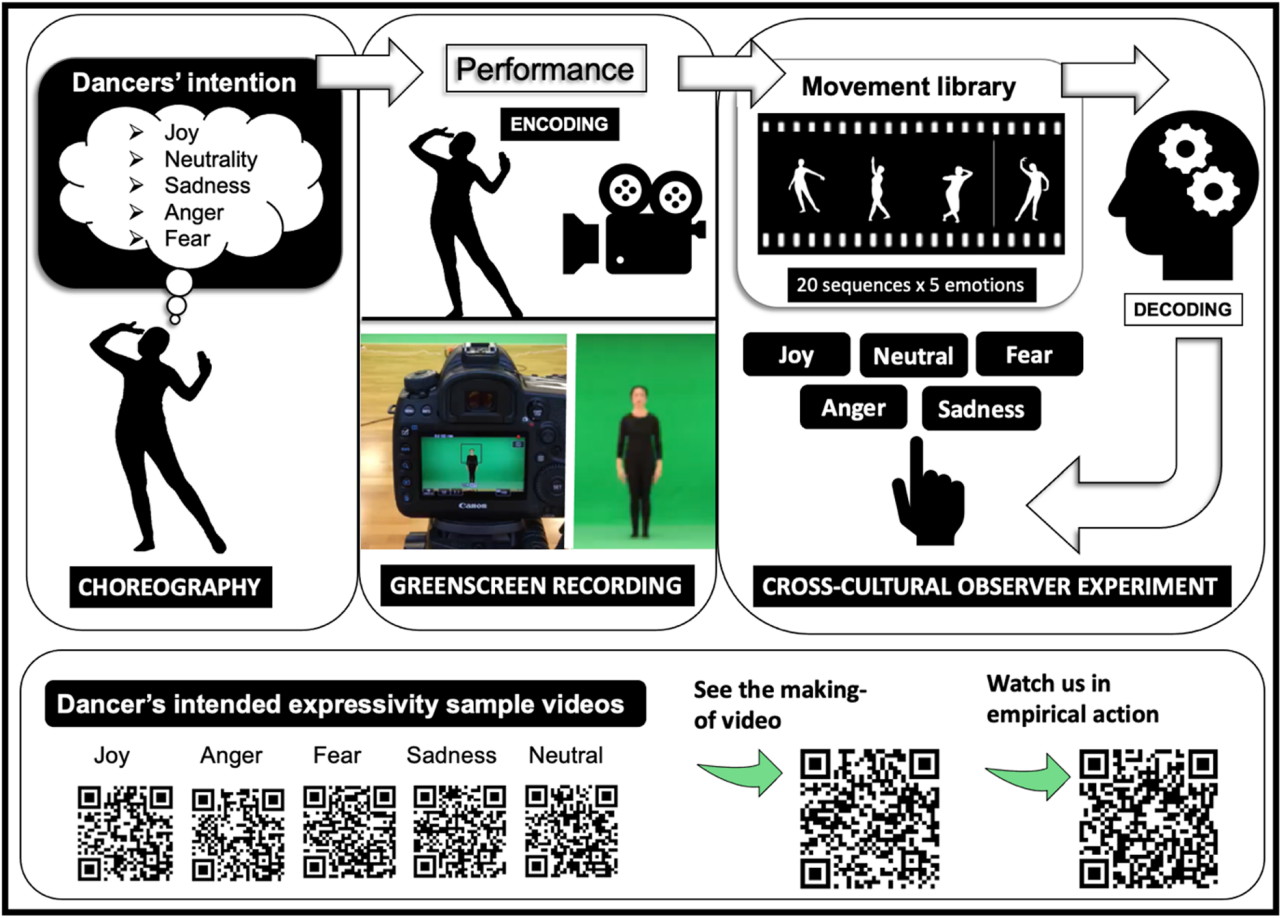
Stimuli creation—library of Iranian classical dance movements. *Note*: The video stimuli were curated by our Iranian film team from 3fish (3fish.co), with Susana Bravo Serra (www.missisbravofilms.com). A Canon EOS 5D Mark IV camera was used for filming, with a Canon EF 24–105 mm f/4 L IS USM lens (settings: framerate (raw) at 50 fps and framerate (output) at 25 fps. White balance: 5000k, shutter speed: 1/100 s, and ISO:400. Video format: H.264, aspect ratio: 16:9, and resolution: 1920 × 1080). Filming took place in a dance studio of City Sport, City, University of London, in front of a standard 6 × 3 m chroma-key greenscreen background to allow for the creation of silhouette videos and blurred faces in postproduction. Dedo-stage lights (7 dedo heads, dimmers and stands kit) were required to illuminate the entire greenscreen and to minimise shadows. Postproduction was done on a 15-inch MAC Book Pro (2017; Processor: 2.9 GHz Quad-Core Intel Core i7; Memory: 16 GB 2133 MHz LPDDR3; Graphics: Radeon Pro 560 4 GB; Intel HD Graphics 630 1536 MB. The softwares Adobe After Effects 2019 and Adobe Premiere Pro 2019 were used for editing and rendering: All footages were imported into Adobe Premiere Pro and were trimmed to the exact start and end points of the movements and soundtrack removed. Each clip was then rendered into a separate file in an uncompressed format and the title was added, as specified verbally by the dancer during the recording. All rendered files were imported to Adobe After Effects and the ‘Keylight’ effect was added and background removed. The ‘Level’ effect (setting: output black = 255) was added to each clip to colour the extracted foregrounds white (the visible dancer silhouette) (H264 format). Please note that following links or the provided QR codes will take you to external sites (YouTube, Vimeo). The authors take no responsibility for ads or other content available on these sites. Please revise the data protection of each site before deciding to consume the content. The videos were produced by the Dance Your Emotion team (video 1) and the Max Planck Institute for Empirical Aesthetics, Frankfurt/M (video 2). The dancer in the picture is Shahrzad Khorsandi (second author). She has consented in full to the publication of the images in an online open-access publication.

A total of 100 full-body Iranian classical dance stimuli (6s each). They consisted of 20 custom-choreographed movement sequences, based on the Iranian dance movement syllabus by Shahrzad Khorsandi^13^. Figure 4 for examples and the stimuli creation process.

#### Stimuli (experiment 2)

Based on the emotion recognition values from Experiment 1, the five stimuli with the highest recognition rate, regardless of sequence, for each emotion were selected, 25 in total.

### Materials: questionnaires (experiments 1 and 2)

Participants filled in three recently developed screening tools in Farsi and English that measure enculturation and acculturation^48^. These questionnaires were developed based on such enculturation and acculturation measures used in immigration psychology^69,78,84–87^. In addition, participants responded to an aesthetic sensitivity measure and demographics questions.

#### The cultural traditions questionnaire (CTQ) (experiments 1 and 2)

The Cultural Traditions Questionnaire (CTQ) probed for enculturation/acculturation levels with two Iranian and the English cultural traditions each: Christmas and Easter in England, and Yalda and Norouz in Iran. The questions of course do not intend to encompass the full breadth of art of a culture, but we assume that they are suitable indicators for en-/acculturation due to their cultural pervasiveness.

The questionnaire contains 16 items about Cultural Traditions; eight about England and eight about Iran (CTQ-en/ir). Items are answered on a 6-point rating scale from (0) *not at all* to (5) *very much*. In a separate proof of principle study^48^, the Cronbach’s alphas for the two scales in each language ranged from 0.86 to 0.97, suggesting very good internal consistency.

#### The arts engagement in childhood questionnaire (AECQ) (experiments 1 and 2)

The Arts Engagement in Childhood Questionnaire (AECQ) examines the level of exposure to Iranian and English arts during the sensitive enculturation period before the age of 12 years. It contains 18 items in total, 9 items about English and 9 items about Iranian arts engagement. The questions are focused around specific artistic materials (dance, music, paintings) from a specific time period (English Impressionism and Qajar period respectively; approx. 1850–1920) and do not intend to encompass the full breadth of art of a culture. The items were inspired by research in immigration psychology that focusses on an individual’s engagement with TV, sports, and artistic endeavours of a culture, specifically during the sensitive period for enculturation before the chronological age of 12 years^68,70–78^.

Items are answered on a 6-point rating scale from (0) *not at all* to (5) *very much*. In a separate proof of principle study^48^, the Cronbach’s alphas for the two scales in each language ranged from 0.75 to 0.91, suggesting very good internal consistency.

#### The enculturation and acculturation quiz (EAQ) (only experiment 2)

This quiz was added to the other two above questionnaires in experiment 2, to contrast knowledge-based (quiz) and experience-based enculturation and acculturation measures (CTQ and AECQ). The Enculturation and Acculturation Quiz (EAQ)^48^ provides a means to gauge an individuals’ acculturation and enculturation via *knowledge* about specific cultural practice and their cues. It requires participants to choose among a list of items (via a simple tick box system), whether a given item is part of a specific cultural practice. Christmas, Easter, Yalda, and Norouz were again chosen as the cultural traditions to probe for. For example, for English Christmas traditions, the item “fir tree” is a “correct” answer, while “chocolate egg” is not. It is a fast to do task that relies only on binary answers. The design of this task was based on an writer recognition task^110^ as a measure of literature expertise. For scoring, the quiz uses the F1 measure (from information retrieval theory) instead of simple accuracies. F1 is the ratio between true positives (TP, correct answers), false positives (FP), and false negatives (FN) (F1 = 2*TP/(2*TP + FP + FN)). Besides, it disregards true negatives (TN; i.e., participants not selecting anything, which would result in an accuracy of 50%). Instead, F1 gives penalties to falsely recognized items and to missed correct items. The value range is between 0 (no correct answer) and 1 (only correct answers).

### Procedure (both experiments)

With regards to the stimuli creation, the procedure was the following. One professional Iranian dancer danced 20 dance movement sequences five times each (total stimuli number = 100), intending to express one of the traditional five emotions^2^ at each repetition (sad, joy, angry, fear, neutral; omitting disgust and surprise, as suggested by^111^. Table 10 illustrates two examples of stimuli sequences that were danced five times each, while intending to express a different emotion at each repetition.

**Table 10:** Illustration of two movement sequences in the Iranian classical dance library.

Movement/position 1	Movement/position 2	Movement/position 3	Movement/position 4	
(A) Sequence: upper body sequence 1				
			
[Shokufeh]	[Qalammu]	[Nasim]	[Raha]	

Movement/position 1	Movement/position 2	Movement/position 3	Movement/position 4	Movement/position 5
(B) Sequence: upper body sequence 3				
				
[Abshar]	[Runama-Abshar]	[Runama-Raha]	[Shokufeh]	[Nasim]

Two examples the Iranian classical dance stimuli set (namely, upper body sequence 1 (panel A) and upper body sequence 3 (panel B), containing respectively 4 and 5 different movements/positions from the Iranian classical dance syllabus. Right Arm (RA) Shokufeh w/SC-R, Left Arm (LA) Qalammu 5th w/SC-L, Nasim LH to chin, Raha-fntl LA. RA = right arm; SC-R = spinal curve to the right; 5th = arm in 5th position; SC-L = spinal curve to the left; LH = left hand; fntl = in the frontal plane of motion; motion; LA = left arm. Abshar-fntl 3rd to Runama-Abshar × 2, Runama-Raha to 2nd, BA Shokufeh, BH Nasim ↓ fntl = in the frontal plane; × 2 = repeated twice; 2nd = arms in 2nd position; BA = both arms; BH = both hands; ↓ = downward motion. Images from Khorsandi^13^. Reproduced with permission from Shahrzad Khorsandi^13^.

The dancer was left entirely free to interpret the instructions regarding the emotional expressivity. No cues were given (e.g., as ‘sad’ is slow, ‘anger’ and ‘joy’ are fast movements), so the dancer expressed in their own intentional way. Stimuli creation was based on previous dance stimuli sets^15,42,112–114^, ensuring a high level of experimental control^14,115^. The clips show the dancer as a white silhouette on a black background; without facial information, nor costuming, nor music. Each clip was faded in and out and lasted ~ 6 s (the movements corresponded to 8 counts in dance theory).

The procedure for the experiments was the following. Experiments were programmed in the online survey tools Qualtrics© (Experiment) or Limesurvey (Experiment 2), and advertised to participants on Prolific®^107^. Each of the surveys had two parts (results from the second part (aesthetic judgment) is presented elsewhere; Khorsandi et al., *in preparation*). Part 1 consisted of a rating task, namely, to guess the emotion expressed in the dance movements via a forced-choice emotion recognition task (Experiment 1). For Experiment 2, a block design was used where participants were asked to both guess the emotion via a forced-choice emotion recognition task (block 1) and to rate their beauty (block 2; presented in Khorsandi et al., *in preparation*). All stimuli were randomized. For the forced-choice emotion recognition task, five different emotion labels were displayed on the screen (anger, joy, fear, sadness, neutrality), and participants were asked to pick one of those emotions as a guess for which emotion the dancer was currently intending to express.

Part 2 of each survey also included the questionnaires. The *Cultural Traditions:* England vs Iran (CTQ-en/ir), the *Arts Engagement in Childhood:* England versus Iran (AECQ-en/ir) (Experiments 1) and the *Enculturation and Acculturation Quiz (EAQ-en/ir)* (Experiment 2), plus additional demographics questions. At the end of part 2, participants were asked a question that was intended to be used to validate the questionnaires against: “How important is Persian Dance to you?”, which was answered on a scale from 0 (not at all) to 100 (very much). We only use the questionnaire scores from these questionnaires in our analyses. The proof of principle analyses in relation to these screening tools is presented elsewhere^48^.

### Analysis plan

We provide confusion matrices for participants’ emotion recognition accuracies separately for experiments 1 and 2, along with chi-square tests to investigate whether average emotion recognition accuracies were over 20% chance level (5 emotions/100 = 20% chance level). Simple independent t-tests are used to show differences between participants’ enculturation questionnaire scores. Subsequently, Repeated Measures (RM) ANOVAs were conducted for each experiment, with the within-group factor emotion (anger, fear, joy, neutral, sad), the between group factor culture (Iranian, English), and the covariates CTQ-en, CTQ-ir, AECQ-en and AECQ-ir for experiments 1, and for experiment 2, also the new scales EAQ-en and EAQ-ir were added. Šidák-adjusted pair-wise comparisons were performed as applicable.

We used chi-square tests, to investigate the enculturation effects on emotion recognition (prediction 2). For this we defined a ‘joy-bias’ and tested, by means of a chi-square test whether there was a bias to attribute any of the emotions to the videos more than other emotions.

## Supplementary Information

Below is the link to the electronic supplementary material.

Supplementary Material 1

## Data Availability

Data from Experiments 1 and 2 are available at: https://osf.io/2evgt/overview.
