# Supplementary material for: An enculturation-induced joy bias for emotion recognition in full-body-movement
Source: Sci Rep. 2025 Oct 23;15:37163. doi: 10.1038/s41598-025-24332-w (PMC12549998; doi:10.1038/s41598-025-24332-w)
Supplement: Supplementary file 1 — Supplementary Material 1 [file 41598_2025_24332_MOESM1_ESM.docx]

Supplementary material for

**An Enculturation-Induced Joy Bias for Emotion Recognition in Full-Body-Movement**

**Experiment 1 Results – Emotion Recognition**

Although technically impossible, some participant IDs in the Iranian group appeared to have participated more than once. We re-ran the analyses based on the first participation of each prolific ID (English participants = 40; Iranian participants N = 32) and the pattern of results was exactly the same, a part from differences in decimal places; see below.

A repeated measures ANOVA was conducted, with the within-group factor emotion (anger, fear, joy, neutral, sad), the between group factor culture (Iranian, English), and the covariates CTQ-en, CTQ-ir, AECQ-en and AECQ-ir. The dependent variable was percentage of correct responses. There was a main effect of emotion F(4, 264) = 3.246, p = .013, partial *η^2^* = .047, no main effect of cultural background F(1, 66) = .454, p = .503, partial *η^2^* = .007, no interaction between emotion and cultural background F(1, 264) = 1.007, p = .404, partial *η^2^* = .015. None of the interactions between emotion and the questionnaire scales were significant (all ps > .271).

Šidák-adjusted pair-wise comparisons revealed that across all participants, joy had been recognized more accurately (m = 47.8%; SE = 1.8%; 95% CI [44.2%, 51.3%]) than anger (m = 34.4%; SE = 1.9%; 95% CI [30.7%, 38.2%]), fear (m = 31.9%; SE = 1.9%; 95% CI [28.2%, 35.6%]) and neutrality (m = 39.9%; SE = 2.5%; 95% CI [34.9%, 44.8%]) (all ps < .002), while sadness (m = 56.2%; SE = 1.5%; 95% CI [53.2%, 59.1%]) had been recognized over and above all other emotions (all ps < .002). There were no differences in emotion recognition accuracy between neutral and anger (p = .727) and neutral and fear (p = .096).
